# The triglyceride-glucose index, metabolic biomarkers, and olive oil in diabetic patients: Real-world evidence from a propensity-score matched study

**DOI:** 10.1016/j.clinsp.2025.100618

**Published:** 2025-05-11

**Authors:** Ge Zhi-Wen, Hong Zhong-Xin

**Affiliations:** Department of Nutrition, Beijing Friendship Hospital, Capital Medical University, Beijing, PR China

**Keywords:** Olive oil, Biomarkers, Triglyceride-glucose index, Diabetes mellitus

## Abstract

**Objectives:**

Using the Triglyceride-Glucose (TyG) index to categorize patients and to compare the effects of different oils on metabolic biomarkers among diabetic patients.

**Methods:**

A total of 98 diabetic patients were included in this retrospective hospital-based study of which data were obtained from medical and dietary records. The participants were divided into soybean oil groups or olive oil groups according to the dietary packages they chose during hospitalization, which merely differed in the kinds of cooking oil (olive oil vs. soybean oil). After being matched based on propensity scores in a 1:1 ratio with a 0.2 caliber width, 70 subjects were finally included in the analysis.

**Results:**

Compared to admission, blood pressure, lipids, and postprandial blood glucose were decreased at discharge in both groups. Grip strength levels of both hands increased in the olive oil group, while no statistical differences were found for these measures in the soybean oil group. In both the soybean oil and olive oil groups, metabolic biomarkers showed broader improvements at discharge for participants with high TyG levels compared to those with low TyG levels. Additionally, patients with high TyG levels in the olive oil group experienced the greatest benefits in terms of blood glucose, lipids, blood pressure, and handgrip strength before and after hospitalization.

**Conclusions:**

Olive oil may have more advantages in improving metabolic risk factors among diabetic patients. The TyG index can be a valuable tool for clinically screening and categorizing patients for personalized precision treatment.

## Highlights

• Olive oil has more obvious effects in improving muscle strength than soybean oil.
• Patients with high TyG exhibit more improvements in biomarkers to interventions.
• The TyG index is a valuable tool for clinically screening and categorizing patients.
• TyG index can be used for personalized precision treatment of diabetes.

## Introduction

Diabetes mellitus is an important risk factor for cardiovascular morbidity, which is one of the leading causes of disability or mortality worldwide.^1^ The increased risk of developing type 2 diabetes is associated with reduced muscle mass and decreased muscle strength.^2^ Besides, blood pressure, lipid profile, and muscle strength may affect disease progression and prognosis in diabetes, and the mechanisms underlying this multimorbidity coexistence and mutual interaction^3^, ^4^ are not fully defined. Therefore, nutritional treatment for diabetes needs to be considered comprehensively, via maintaining a healthy weight, delaying muscle attenuation, and improving glycaemic control and other metabolic risk factors.

Regarding dietary management, the type of dietary fat is more determinant of outcome than the total amount of fat intake.^5^^,^^6^ An increasing number of studies have begun to focus on unsaturated fatty acids’ role in diabetes management.^7^ An improper ratio of n-6/n-3 Polyunsaturated Fatty Acids (PUFA) may adversely affect blood lipids in diabetic patients, while similar issues have not been identified in Monounsaturated Fatty Acids (MUFA).^8^

The distribution of fatty acids varies in different kinds of cooking oil. Few studies have compared the effects of cooking oils on metabolic risk factors of diabetes.^9^ As a real-world study, this research was conducted to compare the effects of soybean oil, which is rich in PUFA, and olive oil, which is rich in MUFA, on blood pressure, lipids, blood glucose, and handgrip strength among diabetic patients. At the same time, because patients with diabetes are often in a state of multi-morbidity, the authors utilized the Triglyceride-Glucose (TyG) index, which has been validated as an index to predict type 2 diabetes as well as metabolic syndrome, and cardiovascular disease,^10^ to assess the status of insulin resistance in patients. As a convenient and quick index, TyG is hot in predicting the risk of chronic diseases, while there are fewer studies on the practical application of clinical diabetes treatment. Therefore, this study can provide a basis for the application of TyG in the clinical nutritional therapy of diabetic patients.

## Materials and methods

### Study design and participants

This study was a retrospective analysis of data collected from inpatients diagnosed with type 2 diabetes and hospitalized at Beijing Friendship Hospital affiliated with Capital Medical University (Beijing, China) between January 1st and March 31st, 2017. Patients were excluded from the study if they were older than 80, bed-ridden or pregnant, or if they had hyperthyroidism, severe hepatic, renal, or heart-lung failure, myasthenia, coma, moderate-to-severe neurological cognitive impairment, retinal detachment, upper limb fracture or disability.

The patients ordered meals in the hospital during hospitalization, the daily dietary intake, snacks (usually cucumber, tomato, and soda biscuit), and the amount of exercise of the patients were recorded. Participants were optional for two dietary packages, which differed only in the kinds of cooking oil (olive oil vs soybean oil), and other food materials, and the amount of cooking oil was consistent. Each participant in the study consumed one package of oil daily. Each package contained 20*g* of cooking oil. Patients were divided into the Olive Oil (OO) group and Soybean Oil (SO) group depending on the packages they selected.

In total 98 cases without missing information were included in the study, patients who voluntarily chose to eat olive oil packages during hospitalization were matched with those who decided to eat soybean oil packages based on propensity scores in a 1:1 ratio with a 0.2 caliber width. 70 subjects were finally included in the analysis.

The study was established following the ethical guidelines of the Helsinki Declaration and was approved by the Human Ethics Committee of Beijing Friendship Hospital (2015-P2–084–01). Each participant in the study provided his or her written informed consent.

### Data collection

Gender, age, Body Mass Index (BMI), family history of diabetes, duration of diabetes, history of other chronic diseases, medication use, lifestyle habits (smoking and drinking), Length of hospital Stay (LOS), biochemical parameters, dietary intake, and exercise during hospitalization were obtained from medical records and dietary records. Systolic Blood Pressure (SBP), Diastolic Blood Pressure (DBP), blood lipid profile (Total Cholesterol [TC], Triglycerides [TG], High-Density Lipoprotein Cholesterol [HDL-C], Low-Density Lipoprotein Cholesterol [LDL-C]), glycated Hemoglobin (HbA1c), Fasting Blood Glucose (FBG), Postprandial Blood Glucose (PBG), Left Handgrip Strength (L-HGS), and Right Handgrip Strength (R-HGS) were tested at the time of admission and discharge. The triglyceride-glucose (TyG) Index^10^ was calculated as Ln (TG [mg/dL] × glucose [mg/dL]/2).

### Handgrip strength

Handgrip Strength was measured twice for both hands (Xiangshan dynamometer EH101, Zhongshan, Guangdong, China) after admission and before discharge. After explaining the procedure to each patient, HGS measurements were performed and recorded. Patients stood upright and held the dynamometer with their palms inward and the dynamometer displayed outward. The dynamometer did not touch patients’ clothes during measurements. Patients were encouraged to perform a maximal isometric contraction. The maximum data of each hand were recorded respectively.^11^

### Dietary intake and physical activity

Average daily energy intake, and the proportions of protein, carbohydrate, and fat were calculated with Wincome Dietary Management Software (Version 1.0, Shanghai, China).

Exercise intensity was determined by the following criteria: 1) Low intensity: MET < 3; 2) Moderate intensity: 3 ≤ MET ≤ 6; 3) High intensity: 7 ≤ MET ≤ 9; 4) Extremely high intensity: 10 ≤ MET ≤ 11.

### Statistical analysis

The power calculation was performed using the G*Power software program (version 3.1). The authors conducted the calculation of the required sample size with the effect size set at 0.5. At a two-tailed significance of 0.05, the calculation showed that a total of 54 subjects were needed for the study to achieve a statistical test power of 0.95, and the sample size as eventually increased to at least 65, taking into account a 20 % dropout rate.

SPSS 25.0 (SPSS, Inc., Chicago, IL, USA) software was used for all the statistical calculations. Numeric data with normal distribution are presented as means ± Standard Deviation (SD) and compared by *t*-tests. Numeric data with skewed distribution are presented as the median and Inter-Quartile Range (IQR) and compared by Mann-Whitney U *tests*. Categorical variables were presented as count and percentage and compared by χ^2^ test. Propensity score values were estimated by logistic regression using the group as the dependent variable and gender, age, BMI, duration of diabetes, smoking, and drinking as predictors, subjects were matched in a 1:1 ratio with a caliber value defined as 0.2. Results were considered statistically significant when p-values were < 0.05.

## Results

### Participants characteristics

There were no significant differences between groups in demographic characteristics, number of other common chronic diseases, TyG level and diabetes duration at the time of admission. LOS was also not statistically different between the two groups (Table 1).

**Table 1:** Comparison of demographic characteristics between groups at the time of admission.

**Characteristics**	**Soybean Oil Group** **(*n* = 35)**	**Olive Oil Group** **(*n* = 35)**	**χ^2^/t**	***p***
BMI, kg/m^2^	25.80 ± 3.61	25.92 ± 4.83	−0.118	0.906
Age, years	57.94 ± 10.56	58.94 ± 13.90	−0.339	0.736
Gender (Female), n (%)	18 (51.4 %)	12 (34.3 %)	2.100	0.147
Family history of diabetes (Yes), n (%)	21 (60.0 %)	21 (60.0 %)	0.000	1.000
Diabetes duration, years	9.00 (3.00, 20.00)	10.00 (4.00, 15.00)	−0.295	0.768
History of smoking^a^ (Never), n (%)	21 (60.0 %)	17 (48.6 %)	0.921	0.337
History of drinking^a^ (Never), n (%)	27 (77.1 %)	22 (62.9 %)	1.701	0.192
Length of hospital stay, days	8.00 (7.00, 10.00)	8.00 (7.00, 12.00)	−0.635	0.525
Glycated haemoglobin (%)	9.26 ± 2.06	8.59 ± 1.46	1.582	0.118
TyG^a^	9.25 ± 0.62	9.18 ± 0.71	0.424	0.673
Number of chronic diseases	12.60 ± 5.36	13.03 ± 4.56	−0.360	0.720

a History of smoking: No: those who had never consumed alcohol in their lifetime; Yes: those who consumed ≥ 5 cigarettes per day. History of drinking: No: those who had never consumed alcohol in their lifetime; Yes: those who consumed alcohol ≥ 50 mL/d. TyG, Triglyceride-Glucose Index. Results were considered statistically significant at *p* < 0.05.

Besides, the authors observed no significant difference between groups in the daily dietary intake of energy, proportions of protein, carbohydrate, and fat during the hospitalization. Patients took low-intense physical activities like walking (speed < 4.5 km/h, MET < 3) during hospitalization. The average daily exercise duration was not statistically different between groups (Table 2).

**Table 2:** Comparison of nutrient intake, hypoglycemic drugs and hours spent exercising between groups during hospitalization.

**Characteristics**	**Soybean Oil Group** **(*n* = 35)**	**Olive Oil Group** **(*n* = 35)**	***t*/*Z***	***p***
Energy, Kcal	1554.72 ± 61.64	1543.27 ± 63.21	0.767	0.446
PRO%^a^, %	20.09 ± 0.28	20.00 ± 0.42	1.000	0.321
CHO%^a^, %	53.06 ± 1.37	53.09 ± 1.12	−0.095	0.924
FAT%^a^, %	28.43 ± 0.92	28.51 ± 0.85	−0.405	0.687
Exercise, min	27.66 (20.00, 30.00)	30.00 (20.00, 30.00)	−0.637	0.524

a PRO%, Proportion of protein; CHO%, Proportion of carbohydrate; FAT%, Proportion of fat. Results were considered statistically significant at *p* < 0.05.

### Comparison between soybean oil group and olive oil group

As shown in Table 3, SBP, DBP, TC, HDL-C, LDL-C, and PBGs were significantly lower in both groups at discharge compared to admission (*p* < 0.05). Furthermore, the olive oil group demonstrated an extra benefit regarding handgrip strength. Both the left and right handgrip strength levels exhibited a significant increase after hospitalization in the olive oil group (*p* < 0.05). However, in contrast, handgrip strength did not display any noteworthy changes at discharge within the soybean oil group.

**Table 3:** Comparison of clinical examined signs and biochemical parameters between soybean oil and olive oil groups.

**Characteristics**	**Soybean Oil Group (*n* = 35)**				**Olive Oil Group (*n* = 35)**			
	**Admission**	**Discharge**	***t*/*Z***	***p***	**Admission**	**Discharge**	***t*/*Z***	***p***
Left-Handgrip strength, kgF	25.37±7.45	25.57±7.65	−0.835	0.409	26.01±9.02	27.22±8.47	−3.457	0.001^a^
Right-Handgrip strength, kgF	28.70±7.89	28.71±7.71	−0.028	0.978	28.53±9.12	30.29±8.97	−2.812	0.008^a^
Systolic blood pressure, mmHg	130.20±13.07	124.51±9.40	2.425	0.021^a^	133.17±20.94	123.17±10.70	3.319	0.002^a^
Diastolic blood pressure, mmHg	80.71±11.58	73.51±7.96	3.767	<0.001^a^	80.14±12.57	74.37±9.02	3.103	0.004^a^
Total cholesterol, mmoL/L	4.62±1.28	3.99±0.98	2.562	0.015^a^	4.53±1.43	3.77±1.14	3.989	<0.001^a^
Triglycerides, mmoL/L	1.27 (1.04, 2.15)	1.46 (1.05, 1.77)	−0.811	0.417	1.48 (0.96, 2.03)	1.22 (1.06, 1.80)	−1.229	0.219
HDL-C^b^, mmoL/L	1.16±0.22	1.09±0.22	2.394	0.022^a^	1.13±0.21	1.06±0.20	3.097	0.004^a^
LDL-C^b^, mmoL/L	2.59±0.92	2.21±0.69	2.233	0.032^a^	2.54±0.89	2.03±0.75	3.641	<0.001^a^
FBG^b^, mmoL/L	8.94±2.13	8.43±2.21	1.368	0.180	8.45±2.12	8.34±1.76	0.428	0.671
PBG1^b^, mmoL/L	13.04±4.40	10.82±3.28	3.056	0.004^a^	12.31±3.76	10.37±2.41	2.619	0.013^a^
PBG2^b^, mmoL/L	12.35±4.19	9.53±3.28	4.276	<0.001^a^	11.55±4.85	9.25±2.74	2.766	0.009^a^
PBG3^b^, mmoL/L	11.62±4.45	8.01±2.72	4.209	<0.001^a^	11.99±5.36	9.26±2.63	3.026	0.005^a^
PBG4^b^, mmoL/L	11.57±2.99	8.85±1.75	4.660	<0.001^a^	11.42±4.35	9.72±2.92	2.680	0.011^a^
PBG5^b^, mmoL/L	13.49±4.75	9.27±2.50	5.680	<0.001^a^	12.55±4.37	9.60±3.17	3.199	0.003^a^
PBG6^b^, mmoL/L	9.85±3.27	8.19±2.87	3.280	0.002^a^	9.92±3.57	9.41±3.53	0.636	0.529
TyG	9.25±0.62	8.97±1.03	1.502	0.142	9.18±0.71	9.08±0.61	1.070	0.292

a Parameters tested at discharge differed significantly from those tested at admission, *p* < 0.05.

b HDL-C, High-Density Lipoprotein Cholesterol; LDL-C, Low-Density Lipoprotein Cholesterol; FBG, Fasting Blood Glucose; PBG1, Blood Glucose 2 h after breakfast; PBG2, Blood Glucose before lunch; PBG3, Blood glucose 2 h after lunch; PBG4, Blood glucose before dinner; PBG5, Blood glucose 2 h after dinner; PBG6, Blood glucose at 10pm.

### Subgroup analysis

The median TyG of the patients in the soybean oil group and the olive oil group at the time of admission was 9.12 and 9.31, respectively. According to this, the subjects of the two groups were further subdivided, those greater than or equal to the median of each group were defined as high level, and those lower than the median were determined to be low level. The subjects were divided into four groups, namely High TyG in SO, Low TyG in SO, High TyG in OO, and Low TyG in OO (Table 4, Table 5).

**Table 4:** Comparison of clinical examined signs and biochemical parameters between subgroups of soybean oil.

**Characteristics**	**Low TyG in SO (*n* = 17)**				**High TyG in SO (*n* = 18)**			
	**Admission**	**Discharge**	***t*/*Z***	***p***	**Admission**	**Discharge**	***t*/*Z***	***p***
Left-Handgrip strength, kgF	25.45±7.82	26.04±8.25	−2.128	0.049^a^	25.29±7.32	25.13±7.24	0.416	0.683
Right-Handgrip strength, kgF	29.65±7.92	30.13±7.30	−1.363	0.192	27.79±7.99	27.37±8.05	0.599	0.557
Systolic blood pressure, mmHg	126.29±11.22	125.29±8.89	0.334	0.743	133.89±13.92	123.78±10.06	3.041	0.007^a^
Diastolic blood pressure, mmHg	77.82±11.63	73.29±7.97	1.398	0.181	83.44±11.16	73.72±8.17	4.800	<0.001^a^
Total cholesterol, mmoL/L	4.22±1.02	3.86±0.99	1.147	0.268	4.99±1.41	4.10±0.99	2.364	0.030^a^
Triglycerides, mmoL/L	1.04 (0.82, 1.24)	1.38 (0.96, 1.64)	−2.107	0.035^a^	2.01 (1.62, 2.83)	1.51 (1.21, 2.13)	−2.504	0.012^a^
HDL-C^b^, mmoL/L	1.18±0.23	1.12±0.22	1.534	0.145	1.15±0.21	1.05±0.22	1.845	0.083
LDL-C^b^, mmoL/L	2.34±0.80	2.13±0.72	0.952	0.355	2.82±0.99	2.29±0.67	2.109	0.050
FBG^b^, mmoL/L	8.05±1.80	8.16±2.25	−0.214	0.834	9.78±2.12	8.68±2.22	2.318	0.033^a^
PBG1^b^, mmoL/L	12.42±4.10	10.78±2.57	1.758	0.098	13.63±4.70	10.86±3.92	2.482	0.024^a^
PBG2^b^, mmoL/L	11.46±2.92	9.44±2.44	3.525	0.003^a^	13.19±5.06	9.61±4.00	3.103	0.006^a^
PBG3^b^, mmoL/L	11.25±4.22	7.19±2.16	3.804	0.002^a^	11.98±4.76	8.79±3.01	2.357	0.031^a^
PBG4^b^, mmoL/L	11.84±3.25	8.84±1.83	3.155	0.006^a^	11.32±2.79	8.87±1.71	3.431	0.003^a^
PBG5^b^, mmoL/L	12.95±4.77	8.98±1.90	3.565	0.003^a^	13.99±4.82	9.55±2.98	4.376	<0.001^a^
PBG6^b^, mmoL/L	8.94±2.93	7.69±2.35	1.410	0.178	10.72±3.41	8.66±3.29	3.871	0.001^a^

a Parameters tested at discharge differed significantly from those tested at admission, *p* < 0.05.

b HDL-C, High-Density Lipoprotein Cholesterol; LDL-C, Low-Density Lipoprotein Cholesterol; FBG, Fasting Blood Glucose; PBG1, Blood Glucose 2 h after breakfast; PBG2, Blood Glucose before lunch; PBG3, Blood Glucose 2 h after lunch; PBG4, Blood glucose Before dinner; PBG5, Blood Glucose 2 h after dinner; PBG6, Blood glucose at 10pm.

**Table 5:** Comparison of clinical examined signs and biochemical parameters between subgroups of olive oil.

**Characteristics**	**Low TyG in OO (*n* = 17)**				**High TyG in OO (*n* = 18)**			
	**Admission**	**Discharge**	***t*/*Z***	***p***	**Admission**	**Discharge**	***t*/*Z***	***p***
Left-Handgrip strength, kgF	26.67±10.81	27.04±10.21	−1.464	0.163	25.38±7.20	27.02±7.31	−3.365	0.004^a^
Right-Handgrip strength, kgF	29.18±10.48	30.11±10.30	−3.698	0.002^a^	27.91±7.88	28.57±8.51	−2.179	0.029^a^
Systolic blood pressure, mmHg	134.24±24.20	126.00±10.55	1.675	0.113	132.17±17.99	120.50±10.41	3.179	0.005^a^
Diastolic blood pressure, mmHg	79.41±11.97	74.71±8.38	1.843	0.084	80.83±13.42	74.06±9.82	2.469	0.024^a^
Total cholesterol, mmoL/L	3.90±0.83	3.51±0.81	1.586	0.132	5.13±1.63	4.18±1.36	4.274	<0.001^a^
Triglycerides, mmoL/L	0.96 (0.83, 1.47)	1.15 (1.04, 1.60)	−1.113	0.266	1.99 (1.61, 2.48)	1.40 (1.11, 2.20)	−2.504	0.012^a^
HDL-C^b^, mmoL/L	1.13±0.17	1.05±0.26	2.149	0.047^a^	1.13±0.25	1.03±0.20	2.191	0.043^a^
LDL-C^b^, mmoL/L	2.13±0.59	1.90±0.55	1.457	0.164	2.93±0.96	2.31±0.86	3.725	0.002^a^
FBG^b^, mmoL/L	7.05±1.15	7.62±1.82	−1.591	0.131	9.77±1.97	9.02±1.44	2.628	0.018^a^
PBG1^b^, mmoL/L	10.60±3.53	9.75±2.16	0.779	0.447	13.93±3.29	10.96±2.55	3.041	0.007^a^
PBG2^b^, mmoL/L	9.31±3.74	8.40±2.65	0.789	0.442	13.67±4.91	10.06±2.65	3.179	0.005^a^
PBG3^b^, mmoL/L	11.50±5.36	8.88±2.85	2.256	0.038^a^	12.46±5.47	9.62±2.43	2.022	0.059
PBG4^b^, mmoL/L	11.09±5.16	9.86±3.49	1.393	0.183	11.73±3.54	9.59±2.34	2.327	0.033^a^
PBG5^b^, mmoL/L	12.54±4.70	9.50±3.65	2.146	0.048^a^	12.57±4.17	9.70±2.75	2.322	0.033^a^
PBG6^b^, mmoL/L	8.89±3.11	10.18±4.45	−1.275	0.221	10.90±3.78	8.69±2.29	1.991	0.063

a Parameters tested at discharge differed significantly from those tested at admission, *p* < 0.05.

b HDL-C, High-Density Lipoprotein Cholesterol; LDL-C, Low-Density Lipoprotein cholesterol; FBG, Fasting Blood Glucose; PBG1, Blood Glucose 2 h after breakfast; PBG2, Blood Glucose before lunch; PBG3, Blood glucose 2 h after lunch; PBG4, Blood Glucose before dinner; PBG5, Blood glucose 2 h after dinner; PBG6, Blood glucose at 10pm.

The results showed that in both the soybean oil and olive oil groups, metabolic biomarkers such as blood pressure, lipids, FBG, and PBG improved more broadly at discharge for participants with high TyG levels compared to those with low TyG levels.

Moreover, among the four groups, the High TyG in OO group experienced the greatest benefits in terms of blood glucose, lipids, blood pressure, and HGS before and after hospitalization. Left and right handgrip strength levels were markedly higher and blood pressure, TC, TG, HDL-C, LDL-C, FBG, PBG1, PBG2, PBG4, and PBG5 levels were significantly lower compared to those at admission (*p* < 0.05).

## Discussion

In addition to abnormal glucose metabolism, diabetes patients often experience the presence of obesity, hypertension, disorders in lipid metabolism, and muscle attenuation. These metabolic risk factors interfere with each other, leading to insulin resistance and dysregulated glucose metabolism in diabetic patients.^12^ Therefore, it is crucial to employ comprehensive interventions to effectively regulate blood glucose levels, enhance blood pressure, lipids, and other metabolic biomarkers, preserve muscle mass and strength, and decelerate muscle deterioration. These interventions play a significant role in determining the overall prognosis of individuals living with diabetes.

In this study, patients in both groups showed significant improvement in blood pressure, TC, HDL-C, LDL-C, and PBG before and after hospitalization, indicating that proper clinical treatment and healthy dietary patterns are important for improving the metabolic disorder of diabetes.^13^ However, FBG did not change significantly, the possible reasons are: first, the improvement in postprandial glucose was more pronounced because of the relatively healthy diet adopted during hospitalization, but the impaired fasting glucose regulation could not be corrected temporarily for a short hospitalization period. In many studies, it often takes a few weeks or more for FBG to show statistical differences.^14^^,^^15^ Second, the relatively tight control of additional meals during hospitalization and prolonged fasting after dinner triggered rebound hyperglycaemia, also known as the Somogyi effect,^16^ increasing blood glucose in the early morning.

In the lipid profiles of the patients in both groups, there was a notable decrease in the other three parameters including TC, HDL-C, and LDL-C, except for TG, which did not change significantly before and after hospitalization. Possible mechanisms include that clinical consideration of cardiovascular risk in people with high TC often leads to pharmacologic interventions such as statins, whereas non-pharmacologic interventions such as dietary control and exercise interventions are often the first to be implemented in patients with increased serum TG levels, and it often takes a few months or more for the effects to become apparent.^17^ Thus, there may not be a significant difference in TG before and after short-term hospitalization.

Moreover, there was a remarkable elevation in handgrip strength levels before and after hospitalization in the olive oil group, but this change was not observed in the soybean oil group, suggesting that olive oil may have a more rapid and obvious effect in improving muscle strength of patients with diabetes than soybean oil. Previous reports have demonstrated the beneficial effects of olive oil on muscle function and strength.^18^^,^^19^ Diabetes can cause muscle dysfunction and decreased muscle strength in patients, mainly through the following mechanisms: (i) Inflammation. Some studies have demonstrated that increased levels of pro-inflammatory cytokine caused by diabetes (e.g., IL-6, TNF-α) inhibit muscle protein synthesis, promote protein degradation, and impair muscle function, which are inversely associated with muscle strength.^20^^,^^21^ (ii) Insulin resistance, which can suppress the insulin/IGF-1 signalling pathway, inhibit protein anabolism, and/or promote protein degradation, relates to the impairment of muscle strength.^22^^,^^23^ (iii) Oxidative stress. Hyperglycaemia, increased intermuscular fat, and insulin resistance may induce mitochondrial dysfunction and stimulate oxidative stress in the organism to produce reactive oxygen species, leading to DNA damage and oxidative damage to proteins, accelerating skeletal muscle cell destruction, and impaired skeletal muscle production.^24^ The anti‐inflammatory and antioxidant features of MUFA and other phytochemicals,^25^ which are abundant in olive oil, may protect against high oxidative stress and insulin resistance, alleviate lipotoxicity in the body and reduce the number of lipid cells stored in adipose tissue, which in turn contributes to reduced muscle attenuation.^26^^,^^27^ Metabolic factors such as blood pressure and serum lipids, as well as handgrip strength, a proxy measure of muscle function and muscle strength, interact with the incidence, progression, and outcome of this diabetes.^3^^,^^4^ The mechanisms underlying this are intersecting and closely related in terms of insulin resistance, inflammation, and oxidation. When exploring the relationship between nutrients and diabetes, more indicators need to be included for comprehensive evaluation, which may be more meaningful for the prevention and treatment of diabetes. As to why the soybean oil group did not show significant differences in handgrip strength levels before and after hospitalization, this may be related to the higher content of n-6 unsaturated fatty acids in soybean oil. Some studies^28^^,^^29^ have found that high intake of n-6 PUFA or excessive n-6/n-3 PUFA ratio is associated with increased BMI, waist circumference, and body fat, and also correlates with levels of pro-inflammatory factors, which in turn exacerbate inflammation levels. In vitro models, studies have found that n-6 PUFA could alter DNA methylation profiles,^30^ which have been related to increased levels of inflammation. Conversely, lowering the n-6/n-3 ratio contributed to improving lipid and glucose metabolism.^31^^,^^32^

The TyG index, calculated by fasting Triglycerides (TG) and blood glucose levels, has been proposed as a reliable indicator for identifying insulin resistance across various populations.^33^^,^^34^ A significant correlation between TyG and glucose metabolism has been reported. Due to its simplicity and cost-effectiveness, TyG has gained popularity in the prediction and large-scale screening of prediabetes and diabetes.^10^^,^^33^ However, its use in the clinical management of diabetic patients is less common, and further research is necessary. The findings of this study suggest that diabetic patients with elevated TyG levels exhibit increased responsiveness to interventions, leading to significant improvements in metabolic biomarkers such as blood pressure, lipids, glucose levels, and handgrip strength during hospitalization periods of similar duration. Santiago's research also highlighted that individuals with pre-diabetes and higher TyG index independently experienced greater reductions in diabetes-related markers like BMI, FBG, and TG through lifestyle interventions.^35^ Prior research has found that controlled glycemia was associated with improved CV outcomes in diabetic patients, especially in those with high TyG index levels.^36^ Considering the positive correlation between higher TyG levels and poor prognosis,^37^^,^^38^ increased cardiovascular disease risk,^36^^,^^39^ and even mortality risk,^40^ as well as the more pronounced therapeutic effects, clinical treatment can begin by stratifying patients and tailoring interventions that are more suitable for achieving greater effectiveness.

As a real-world study, there were inherent limitations, such as small sample size, non-random distribution of patients between groups, interference with clinical treatment, and lack of health control. Also, because there are few studies about the effect of vegetable oils on handgrip strength improvement in diabetics, the power calculation used fixed parameters, not experimental data from one of the previous studies, the results of the primary outcome will be used for sample size estimation in a future study. The main strength of the present study was that the inpatients were all served uniformly, with both packages differing only in cooking oil, controlling for the interference of other dietary factors. Plus, participants were propensity score matched to improve study quality.

## Conclusion

In conclusion, when comparing soybean oil to olive oil, it appears that olive oil may have a greater effectiveness in enhancing muscle strength among individuals with diabetes. This, in turn, could offer potential benefits by reducing insulin resistance, slowing down the progression of diabetes, and enhancing overall prognosis. Furthermore, in terms of diabetes interventions, patients with elevated TyG levels tend to experience more substantial and noteworthy improvements in blood pressure, lipids, glucose, and muscle strength. Consequently, the TyG index can serve as a valuable tool for clinically screening and categorizing patients for personalized precision treatment.

## Ethical statement

The study was approved by the Ethics Committee of Beijing Friendship Hospital (2015-P2–084–01) and followed the STROBE Statement. Each participant in the study provided his or her written informed consent.

## Authors’ contributions

Ge Zhi-Wen: Conception and design of study, analysis and interpretation of data, and preparation of manuscript.

Hong Zhong-Xin: Constructive discussions.

All authors have read and approved the final version of the manuscript.

## Declaration of competing interest

The authors declared no potential conflicts of interest concerning the research, authorship, and/or publication of this article.
